# A Universal Framework for General Prediction of Physicochemical Properties: The Natural Growth Model

**DOI:** 10.34133/research.0510

**Published:** 2024-10-23

**Authors:** Jinming Fan, Chao Qian, Shaodong Zhou

**Affiliations:** ^1^ College of Chemical and Biological Engineering, Zhejiang Provincial Key Laboratory of Advanced Chemical Engineering Manufacture Technology, Zhejiang University, 310058 Hangzhou, P. R. China.; ^2^ Zhejiang Provincial Innovation Center of Advanced Chemicals Technology, Institute of Zhejiang University - Quzhou, 324000 Quzhou, P. R. China.

## Abstract

To precisely and reasonably describe the contribution of interatomic and intermolecular interactions to the physicochemical properties of complex systems, a chemical message passing strategy as driven by graph neural network is proposed. Thus, by distinguishing inherent and environmental features of atoms, as well as proper delivering of these messages upon growth of systems from atoms to bulk level, the evolution of system features affords eventually the target properties like the adsorption wavelength, emission wavelength, solubility, photoluminescence quantum yield, ionization energy, and lipophilicity. Considering that such a model combines chemical principles and natural behavior of atom aggregation crossing multiple scales, most likely, it will be proven to be rational and efficient for more general aims in dealing with complex systems.

## Introduction

The rapid development of current chemical and materials industries requires rapid and accurate prediction of various properties involving multiple scales. However, the advances of quantum mechanics and classic thermodynamics cannot cover all these scales, which poses a challenge to the rational, ab initio design of higher-performance systems [1]. Thanks to the development of data-driven machine learning approaches [2,3], new energy has been infused into the fields of materials science and chemistry [2,4–10]. Thus, a sufficient number of samples can be used to provide a range of molecular properties through the trained model, which greatly accelerates the discovery of new functional molecules [11–14]. In predicting chemical properties, it is necessary to convert the chemical structure into computer-recognizable form of data. To this end, molecular descriptors [15] and molecular fingerprints [16] have been employed and proven to be useful for predicting various properties. However, these features may not be directly related to the properties of the molecule; they thus do not reflect the nature of interatomic or intermolecular interactions. Recently, machine learning models based on thermodynamic theory have also been used for prediction [17,18], but the complexity of feature processing limits generic application of these models. In any case, a fast and general model for multiscale property prediction is still lacking. Such a model is expected to be established based on chemical principles.

With the development of deep neural networks [19], representation learning has shown great advantages over feature based methods [20], attracting more and more attention concerning molecular property prediction. Graph neural network (GNN) learning for molecular representation has recently become an emerging research field that the topology of atoms and bonds is treated as a graph and propagates messages sent by each element to its neighbors [21,22]. However, although GNN can achieve feature transfer and aggregation from an atomic level, they still require guidance from certain chemical rules for the sake of efficiency and accuracy [23]. Further, in a specific system, the chemical environment is often more complex than the molecule itself. At this point, due to the inherent defects of the molecular graph, it is difficult to consider the chemical environment surrounding the molecule, such as solvent molecules, during message passing. Therefore, it remains challenging how to assign more chemical rules to the message passing process and to consider intermolecular interactions using GNN.

**Fig. 1.**
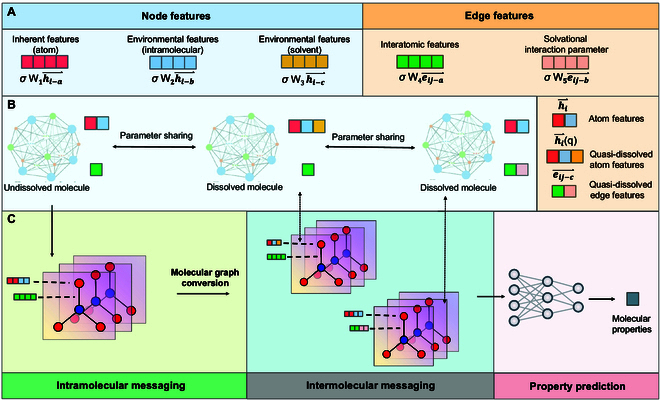
Flow chart of the framework as developed in this work. (A) Multiscale features of 3 molecular graphs. (B) Features of 3 molecular graphs. (C) The working principle of NGNN.

In order to endow GNN with more chemical rules, it is necessary to describe interatomic and intermolecular interactions reasonably. Based on this, more accurate message transmission is possible when subjecting a molecule in chemical environments. For this purpose, we have defined multiscale features (Fig. 1A), which are derived from undissolved molecules and transformed into 2 dissolved molecular graphs through different feature extensions (Fig. 1B). This allows information to gradually diffuse from intramolecular to intermolecular, fundamentally describing the contribution of each atom in the mixed system to the target properties (Fig. 1C). This natural growth neural network (NGNN) harmonizes chemical principles with algorithmic concepts, enabling accurate prediction of complex system properties.

## Results and Discussion

**Fig. 2.**
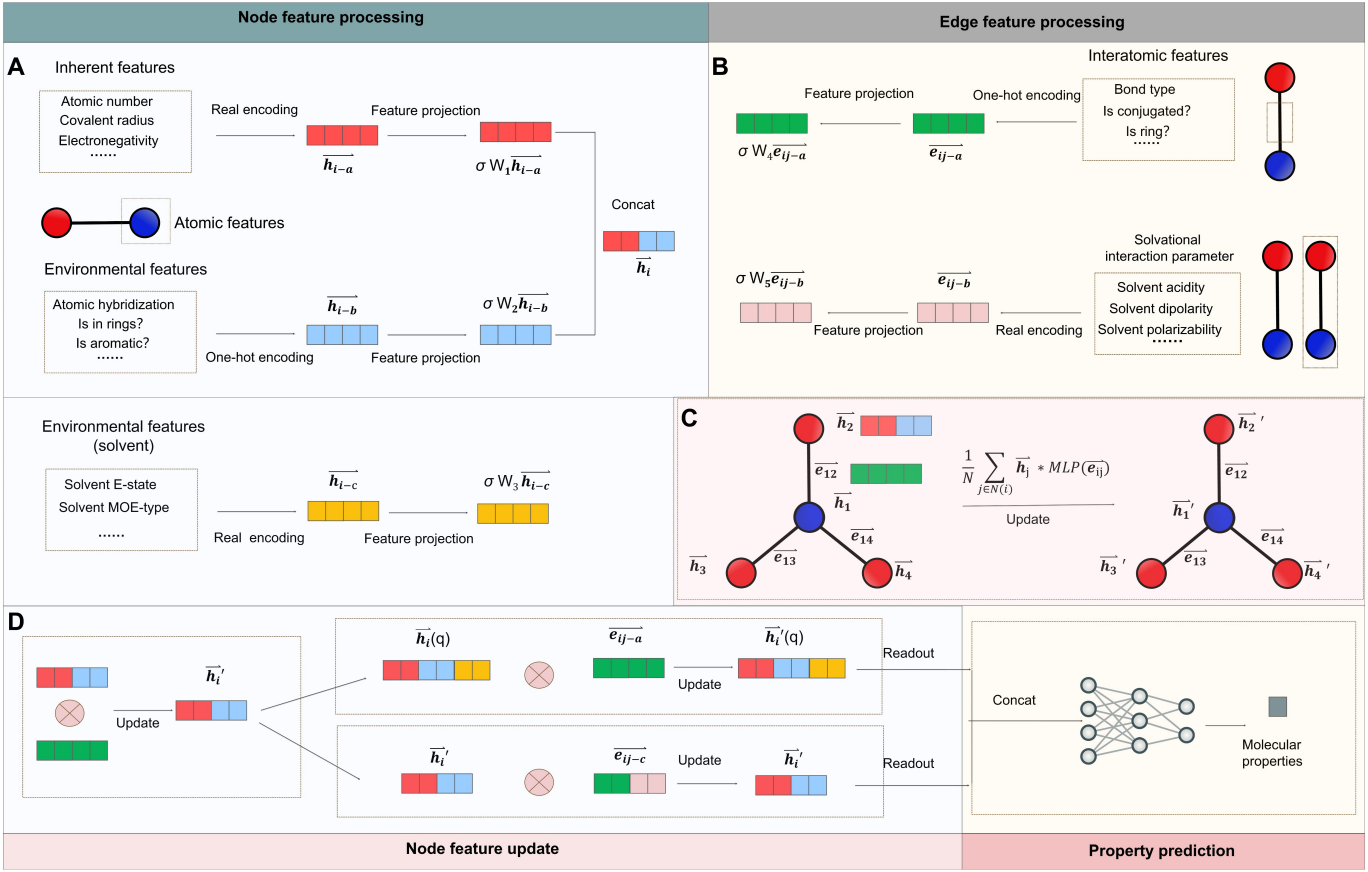
The workflow of NGNN. (A) Encoding and calculation of different node features. (B) Encoding and calculation of different edge features. (C) The way of message passing. (D) The way of multistage message passing between atoms and molecules and property prediction.

GNN often used one-hot coding to encode the chemical environment of atoms, which whereas could not accurately describe the physical meaning of atoms. As shown in Fig. 2A, in order to regulate the model with more chemical rules, quite a few multiscale features have been introduced. For atomic features of specific molecules, they are classified into inherent atomic features ($\overset{⃑}{h_{i-a}}$) and atomic environment features ($\overset{⃑}{h_{i-b}}$). The inherent features are determined by the element itself, while the environmental characteristics are exerted by each molecule. Furthermore, one can also label atoms or groups that have an important impact on the results (such as hydrophobic atoms for prediction of water solubility). Thus, from a chemical perspective, the formulation of these features are incompatible. Further, the inherent features chosen are all encoded in natural form, while the environmental features are encoded in one-hot form. Therefore, these features have to be mapped to separate spaces using different scales ($W_{1}$ and $W_{2}$) to ensure that the extracted features can accurately describe the complete state of each atom in the molecule, and the calculation method is proposed as Eq. 1:

$$\overset{⃑}{h_{i}}=\text{concat}\left(\overset{⃑}{h_{i-a}},\overset{⃑}{h_{i-b}}\right)$$

$$=\text{concat}\left(\sigma \left(W_{1}*\overset{⃑}{h_{i-a}}\right),\sigma \left(W_{2}*\overset{⃑}{h_{i-b}}\right)\right)$$ (1)

in which ***σ*** is activation function. For message passing between atoms, the edge features is used to drive the update of atomic nodes, using Eq. 2:

$${\overset{⃑}{h_{i}}}^{k}=\Theta {\overset{⃑}{h_{i}}}^{k-1}+\frac{1}{N}\sum_{j\in N\left(i\right)}{\overset{⃑}{h_{j}}}^{k-1}*\mathrm{MLP}\left(\sigma \left(W_{4}*\overset{⃑}{e_{\mathrm{ij}-a}}\right)\right)$$ (2)

After node feature updates, the intact molecular graph is transformed into 2 forms of dissolved molecular graphs.

1. The description of solvent topology using electronic features, and the connectivity information and van der Waals radius to calculate the atomic contribution to a target property within the van der Waals surface area [24,25]. Starting from the charge and molecular structure of the solvent, the solvent effects are interpreted from dissolved molecules to quasi-dissolved atoms. Such dissolution of atoms proceeds via Eq. 3:

$$\overset{⃑}{h_{i}}\left(q\right)=\text{concat}\left({\overset{⃑}{h_{i}}}^{\prime },\sigma \;\left(W_{3}*\overset{⃑}{h_{i-c}}\right)\right)$$ (3)

2. Applying the solvent effects on dissolved molecules to atomic interactions including bonding, long-range interactions, and concentration, that is, adding solvational interaction parameter [26,27] to the edge features. The solvent effects on each atom are considered in the course of information passing, as implemented using Eq. 4:

$$\overset{⃑}{e_{\mathrm{ij}-c}}=\text{concat}\left(\sigma \left(W_{4}*\overset{⃑}{e_{\mathrm{ij}-a}}\right),\sigma \left(W_{5}*\overset{⃑}{e_{\mathrm{ij}-b}}\right)\right)$$ (4)

Next, message passing between molecules operates in 2 different ways (Eqs. 5 and 6):

$$\overset{⃑}{h_{i}}{\left(q\right)}^{k}=\Theta \overset{⃑}{h_{i}}{\left(q\right)}^{k-1}+\frac{1}{N}\sum_{j\in N\left(i\right)}{\overset{⃑}{h_{j}\left(q\right)}}^{k-1}*\mathrm{MLP}\left(\sigma \;\left(W_{4}*\overset{⃑}{e_{\mathrm{ij}-a}}\right)\right)$$ (5)

$${\overset{⃑}{h_{i}}}^{k}=\Theta {\overset{⃑}{h_{i}}}^{k-1}+\frac{1}{N}\sum_{j\in N\left(i\right)}{\overset{⃑}{h_{j}}}^{k-1}*\mathrm{MLP}\left(\overset{⃑}{e_{\mathrm{ij}-c}}\right)$$ (6)

After completing the message passing between molecules, the 2 dissolved molecular graphs are output via Eq. 7:

$$\overset{⃑}{h_{G}}=\text{concat}\left(\overset{⃑}{h_{G-1}},\overset{⃑}{h_{G-2}}\right)=\text{concat}\left(\mathrm{Set}\left(\sum_{N\left(i\right)}\overset{⃑}{h_{i}}\left(q\right)\right),\mathrm{Set}\left(\sum_{N\left(i\right)}\overset{⃑}{h_{i}}\right)\right)$$ (7)

in which ***Set*** is a readout operator [28]. Finally, a separate neural network is used to predict molecular properties (Eq. 8:

$$y=\mathrm{MLP}\left(\overset{⃑}{h_{G}}\right)$$ (8)

To demonstrate the ability of the NGNN model to handle intermolecular interactions, 3 single-solvent datasets and 3 multisolvent datasets were employed for test with quite a few physicochemical properties as the target features, including the adsorption wavelength, emission wavelength, solubility, photoluminescence quantum yield (PLQY), ionization energy (IE), and lipophilicity (for more details, see Materials and Methods).

As shown in the previous text, we have designed a universal model using features that can essentially describe the inherent characteristics of each atom and the impact of chemical environment on the target properties. Through the transfer and aggregation of features between atoms, we ultimately obtain the molecular properties of specific environments, without the need to design specially targeted atomic and environmental features. On the other hand, for some functional groups or atoms sensitive to the target property, they can be marked out when describing the environmental characteristics. For example, the conjugated fragments can be labeled when predicting the spectroscopic properties, while the likely formed hydrogen bond should be considered when designing the features for predicting azeotropic points. As a more explicit instance, in predicting water solubility, we can label hydrophobic groups (such as F, Cl, Br, and I) in environmental features $\left(\overset{⃑}{h_{i-b}}\right)$. As shown in Table 1, the labeled model exhibits higher accuracy than the unlabeled one.

**Table 1.** The average performance of solubility in 3 independent runs

Method	MAE	*R* ^2^
NGNN-unmarked	0.4410	0.8500
NGNN-marked	0.3887	0.8791

**Fig. 3.**
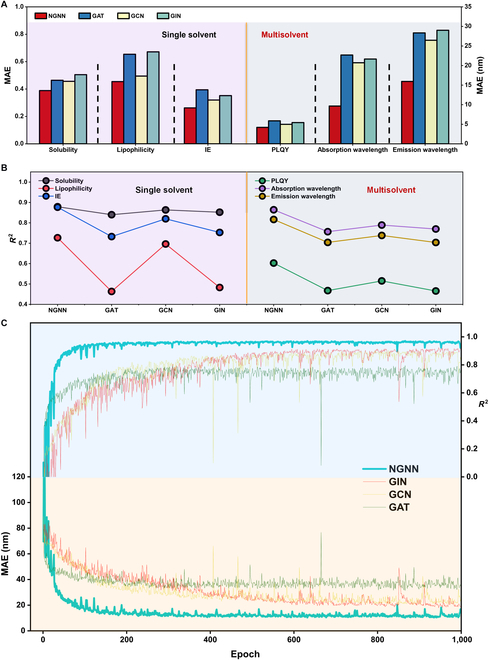
Prediction of physicochemical properties with different models. (A) MAE of different models. (B) *R*^2^ of different models. (C) Training curves of different model in the prediction of absorption wavelength.

As shown in Fig. 3A and B, compared with other benchmark GNNs (graph convolutional network [GCN], graph attention network [GAT], and graph isomorphism network [GIN]) [29–31], NGNN exhibits high superiority. In the prediction of single-solvent data, the excellent performance of NGNN indicates that the interatomic message passing logic is indeed advanced in describing the interatomic and intermolecular interactions, in comparison with simple data stacking. Furthermore, in the prediction using multisolvent datasets, although with much smaller data volume and higher complexity (12 solvents), the superior performance of NGNN exhibits extremely high homogeneity for training set and prediction set (Fig. 3C).

**Fig. 4.**
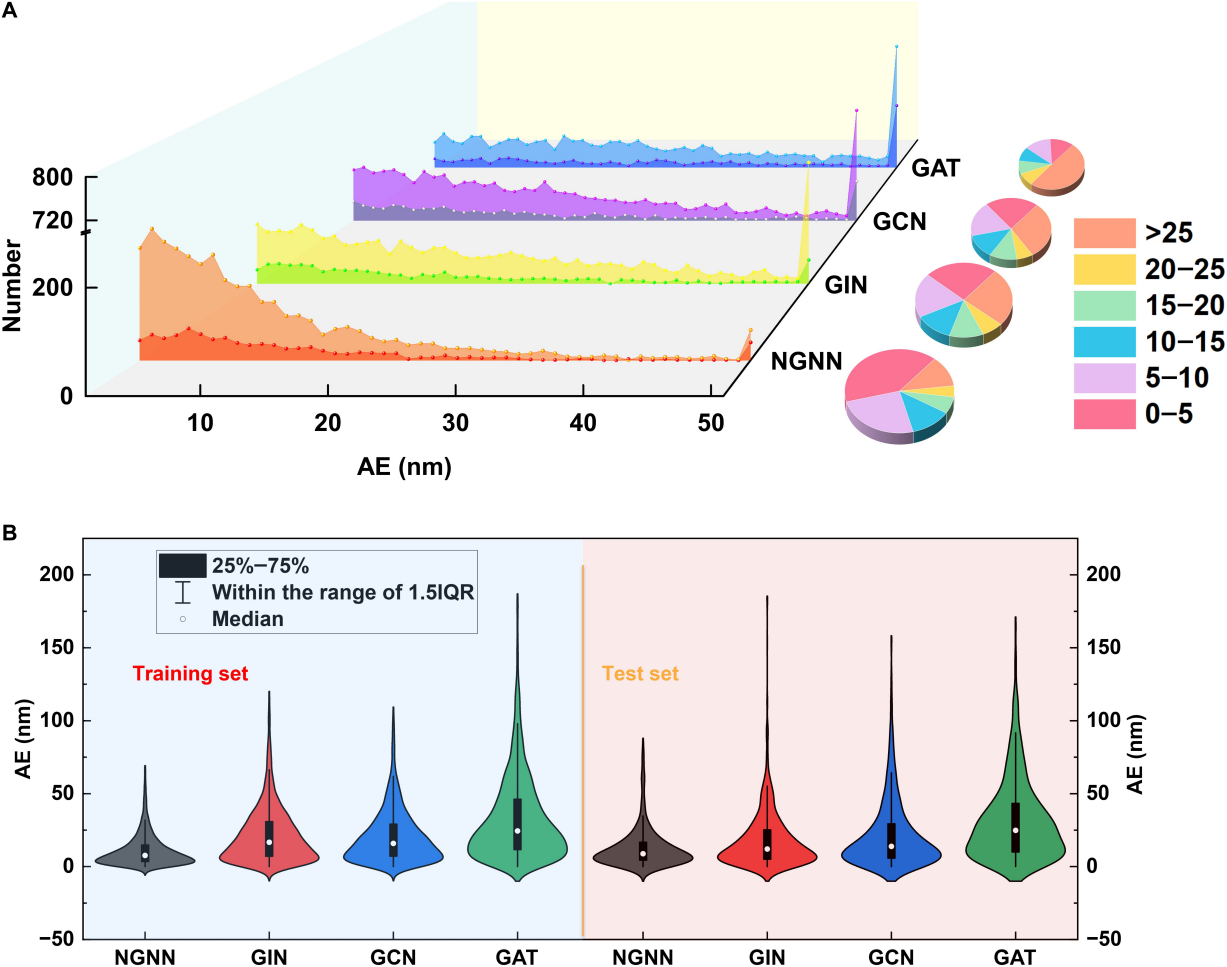
The robustness of different models in the prediction of absorption wavelength. (A) Absolute error (AE) distribution of different models. (B) Absolute error distribution of different model training and test sets.

Next, the prediction of adsorption wavelength was employed to examine the robustness of different models. As shown in Fig. 4, NGNN is of stronger stability as compared to the previously reported models, and the errors are insignificant for both training and prediction. This indicates that NGNN can better understand the interactions within and between molecules and rationally apply them to unknown molecular predictions. Clearly, the message passing strategy proposed in this work can integrate the information of different solvent molecules with the characteristics of dissolved atoms and edges, thereby learning the influence of interconstituent interactions crossing multiple scales. Considering the higher dependency of absorption wavelength on both interatomic and intermolecular interactions, most likely, the NGNN model touches the essence of mixing.

**Fig. 5.**
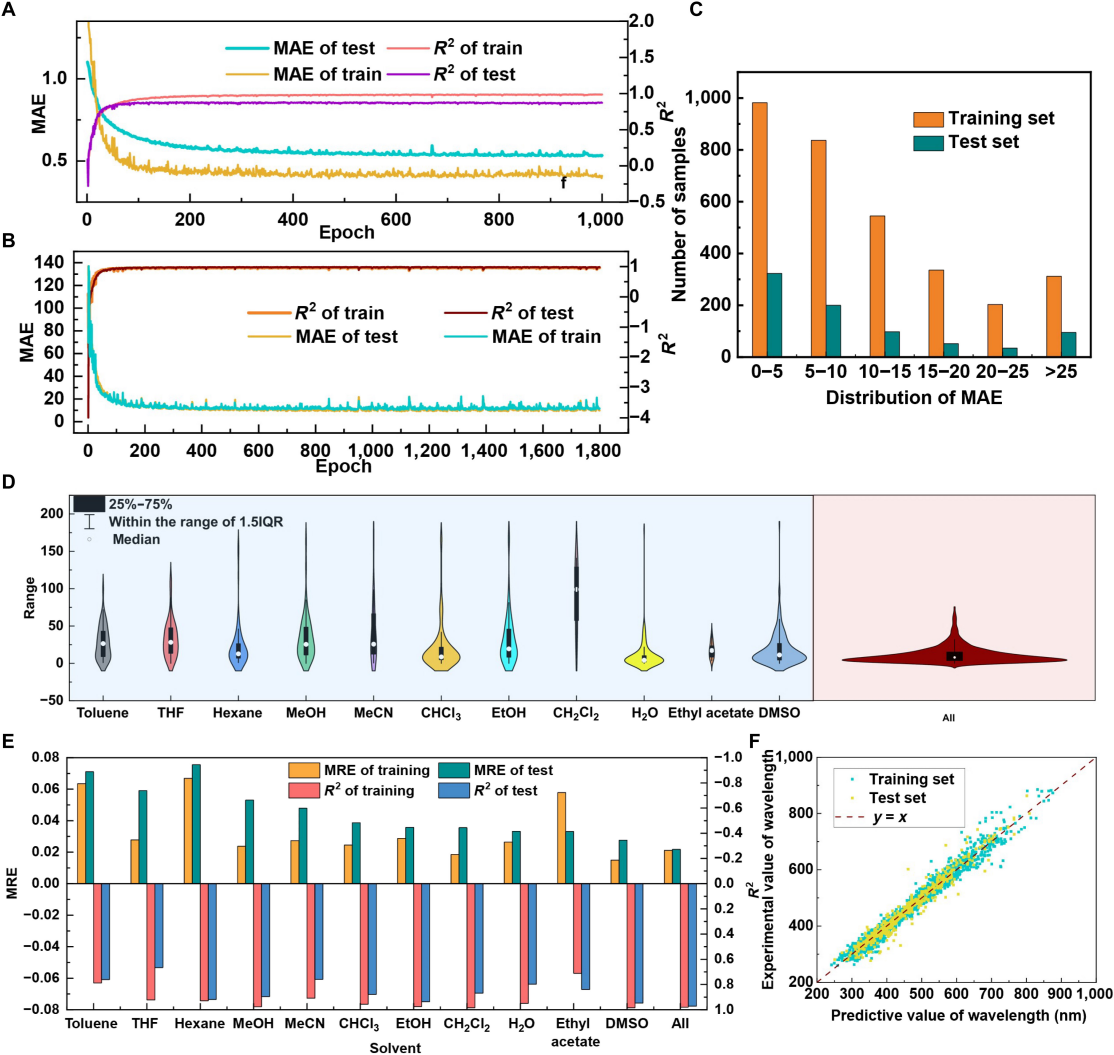
Prediction results of solubility and absorption wavelength with different solvents using NGNN. (A) Training curves of NGNN in the prediction of solubility. (B) Training curves of NGNN in the prediction of absorption wavelength. (C) The distribution of MAE in the prediction of absorption wavelength. (D) Violin plot of MAE distribution under different solvent data in absorption spectrum prediction. (E) Prediction results for different solvents. (F) The correlation between the predicted and experimental values of the absorption wavelength.

Further, to track the source of error, the error changes were traced all during the learning process. As shown in Fig. 5, the error changes for the train set evolve consistently with that for the test set in the modeling of absorption wavelength (Fig. 5B), which further proves the advance of the NGNN model in rationality. On the other hand, for water solubility prediction, due to the data available for only one solvent, the model relying solely on the properties of the solute itself, resulting in significant overfitting (Fig. 5A). Moreover, as shown in Table 2, traditional machine learning models (random forest [RF] and gradient boosting regression tree [GBRT]) [32,33] based on statistics lack guidance from chemical rules. To obtain better performance in the test, overfitting of the training set is unavoidable. In contrast, by considering the interatomic and intermolecular interactions from multiple perspectives, and through reasonable feature aggregation and propagation, NGNN is able to describe the characteristics of the system even upon scaling up from atomic level to bulk mixture. The macroscopic properties of a complex system can eventually be derived without obvious overfitting of the model.

**Table 2.** The average performance of different models in predicting absorption wavelengths in 3 independent runs

	Training set				Test set			
Model	MAE	RMSE	MRE	*R* ^2^	MAE	RMSE	MRE	*R* ^2^
GCN	18.6570	27.1291	0.0420	0.9260	21.1936	35.4761	0.0477	0.8709
GIN	24.5175	33.6847	0.0557	0.8796	23.3730	37.0351	0.0523	0.8535
GAT	29.8693	43.9367	0.0675	0.7937	30.9669	46.0222	0.0691	0.7765
NGNN	10.7600	15.8400	0.0236	0.9800	10.6500	18.3300	0.0237	0.9600
GBRT	6.8296	9.8413	0.0160	0.9905	14.1696	25.0745	0.0324	0.9368
RF	4.8454	9.4383	0.0109	0.9912	13.0689	27.1370	0.0299	0.9258

To further demonstrate the behavior of NGNN, we separately modeled each solvent in the absorption wavelength dataset, and the results are shown in Fig. 5. There was a significant overfitting in the test set for each solvent prediction, which is consistent with the trend in the prediction of water solubility. On the contrary, in the case of unified modeling of all data, the errors of the training and testing sets show good consistency. Moreover, the mean absolute error (MAE) distribution (Fig. 5C to E) and correlation (Fig. 5F) of the training and testing sets further demonstrate the high generalization ability of NGNN. Due to previous data limitations (lack of data for solution properties of different concentrations), concentration was not taken into account in the feature. However, this feature can be considered by adding an $\overset{⃑}{e_{\mathrm{ij}-b}}$ term to modify the effect of solute–solvent ratio on the results. If the required data is available, most likely, the solution properties of different concentrations can be afforded.[Fig F6]

**Fig. 6. F6:**
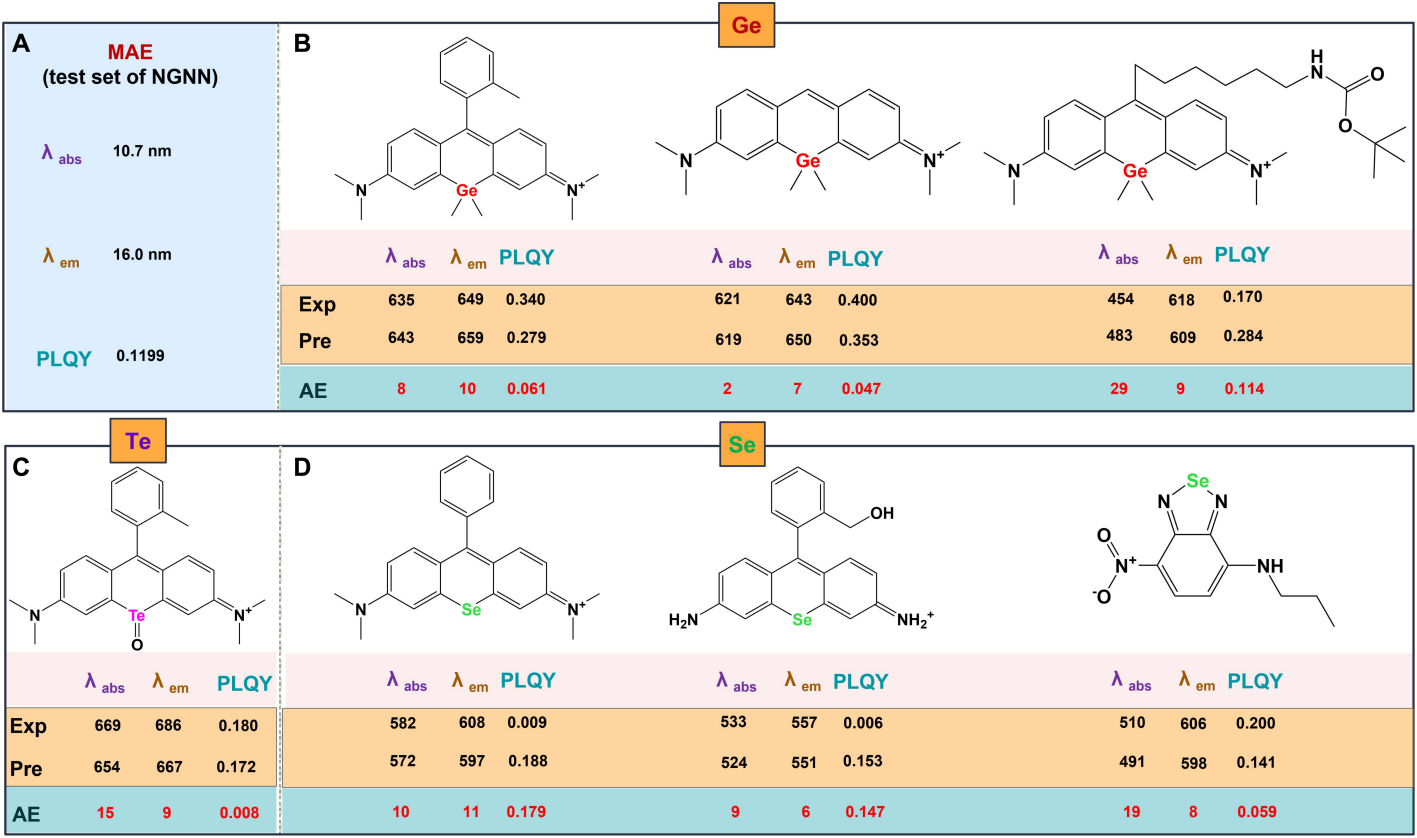
Prediction results of 7 molecules with unprecedented elements used for testing. (A) MAE of NGNN model test set for comparison. (B) Prediction results containing Ge element molecules. (C) Prediction results containing Te element molecules. (D) Prediction results containing Se element molecules.

The design intention of our model is to extract the commonalities of each atomic feature as much as possible to gradually extend it to the entire molecule and complex environment. Thus, in order to better reflect the generalization of the model, we conducted extrapolation test on 7 external molecules, including 3 elements that do not exist in the training set. As shown in Fig. 6, the results showed that although our model have not learned molecules containing these elements, it is able to extract commonalities from the inherent features of atoms and expand them to unknown elements, which may greatly benefit the design of new molecules.

## Conclusion

In summary, we have developed a NGNN framework for general, efficient prediction of a series of physicochemical properties based on proper handling of interatomic and intermolecular interactions of the system. The construction of this framework is based on strict chemical rules and natural behavior of system growing from atomic to bulk levels. By properly describing and transforming atomic features, message passing upon aggregating atoms to complex systems becomes more precise and efficient. Further, solvent features are embedded into molecular graphs in different ways through feature sharing, enabling GNN to handle complex intermolecular interactions. Such a framework is expected to compensate the shortcomings of GNN in predicting the properties of complex mixtures and may therefore be proven to be universally applicable in wider range of chemical/physical aims.

## Materials and Methods

### Data and processing

Water solubility dataset is a curated collection of the aqueous solubilities of organic compounds from 3 literature-based large databases: (a) Vermeire’s [34], (b) Boobier’s [35], and (c) Delaney’s [36]. The produced dataset was prepared by omitting the nonunique measures and noisy data, consisting of more than one solubility measure for a single molecule, yielding a total of 8,438 unique data entries [37]. Detailed information about IE and lipophilicity databases can be obtained on our GitHub website.

As shown in Tables 3 to 5, the absorption wavelength dataset, emission wavelength dataset, and PLQY dataset contain data for 12 solvents. The datasets are from the website (http://www.chemfluor.top).

**Table 3.** The amount of data in the absorption wavelength database under different solvents

Solvent	Toluene	THF	MeOH	MeCN	Hexane	H_2_O
Number	254	243	314	289	98	623
Solvent	EtOH	Ethyl acetate	DMSO	DMF	CHCl_3_	CH_2_Cl_2_
Number	280	52	648	86	232	900

**Table 4.** The amount of data under different solvents in the PLQY database

Solvent	Toluene	THF	MeOH	MeCN	Hexane	H_2_O
Number	232	200	203	258	80	219
Solvent	EtOH	Ethyl acetate	DMSO	DMF	CHCl_3_	CH_2_Cl_2_
Number	143	47	354	68	198	821

**Table 5.** The amount of data under different solvents in the emission wavelength database

Solvent	Toluene	THF	MeOH	MeCN	Hexane	H_2_O
Number	268	243	314	289	98	623
Solvent	EtOH	Ethyl acetate	DMSO	DMF	CHCl_3_	CH_2_Cl_2_
Number	280	53	647	86	212	921

### Method

#### Atomic inherent features ($\overset{⃑}{h_{i-a}}$)

1. Covalent radius.
2. Electronegativity.
3. Atomic number.
4. Atomic mass.
5. First IE.
6. Electron affinity.

Note: These features are all encoded using natural coding.

#### Atomic environmental features ($\overset{⃑}{h_{i-b}}$)

1. Valence: (0, 1, 2, 3, 4, 5, 6).
2. Number of H atoms: (0, 1, 2, 3, 4).
3. Formal charge: (−1, −2, 1, 2, 0).
4. Hybridization: (“s”, “sp”, “sp2”, “sp3”).
5. Is inRing?: (1, 0).
6. Is aromatic?: (1, 0).
7. Custom features. Note: This feature can be defined based on the nature of the target and adopt one-hot coding.

#### Atomic environmental features (solvent) ($\overset{⃑}{h_{i-c}}$)

1. Electrotopological state (E-state) descriptors: This index combines the electronic states of intramolecular bonding atoms and their topological properties in the whole molecular skeleton. According to this descriptor, 3 internal states of the molecular substructure within the molecule are quantified: its element content, its valence state (electronic organization), and its topological state relative to its atomic neighbor [24].
2. Molecular operating environment (MOE-type) descriptors: The MOE-type descriptors use connectivity information and van der Waals radii to calculate the atomic van der Waals surface area contribution of an atom-type to a given property, including polarizability, direct electrostatic interaction, and other factors [25].
3. Topological descriptors: According to this descriptor, the connection state of each atom is used to calculate the exponent, thus providing a highly unique exponent for a given molecule [38].
4. Connectivity descriptors.

Note: These features are all encoded using natural coding. This article utilizes 66 of the aforementioned features.

#### Interatomic features ($\overset{⃑}{e_{\mathrm{ij}-a}}$)

1. Bond type: (“single”, “double”, “triple”, “aromatic”).
2. Conjugated: (1, 0).
3. Aromatic: (1, 0).
4. Ring: (1, 0).

Note: These features are all encoded using one-hot encoding.

#### Atomic features ($\overset{⃑}{e_{\mathrm{ij}-b}}$)

1. Et30 [26].
2. SP: Polarizability [27].
3. SdP: Dipolarity [27].
4. SA: Acidity [27].
5. SB: Basicity [27].

Note: This feature can be defined based on the nature of the target.

In each model training and prediction, we randomly select 80% of the data as the training set and the rest as the test set. We use the following indicators to evaluate the prediction performance of the model: mean relative error (MRE), MAE, root mean square error (RMSE), and the coefficient of determination (*R*^2^).

## Data Availability

The data used and more details (including the codes) about the framework are available at https://github.com/Fan1ing/ngnn.
